# Integrating Socio‐Economic and Seasonal Drivers With Feed Innovations to Enhance Smallholder Dairy Production in Gamo and Wolaita Zones, Southern Ethiopia

**DOI:** 10.1002/vms3.70892

**Published:** 2026-03-14

**Authors:** Asrat Ayza, Yisehak Kechero, Ajebu Nurfeta, Dereje Andualem

**Affiliations:** ^1^ Department of Animal Science College of Agriculture Wolaita Sodo University Wolaita Sodo Ethiopia; ^2^ Department of Animal Science College of Agricultural Sciences Arba Minch University Arba Minch Ethiopia; ^3^ School of Animal and Range Sciences College of Agriculture Hawassa University Hawassa Ethiopia; ^4^ Department of Animal Science College of Agricultural Sciences Dilla University Dilla Ethiopia

**Keywords:** alternative supplements, feeding practices, milk yield, seasonal feed availability, smallholder dairy

## Abstract

**Background:**

Smallholder dairy farming in southern Ethiopia, particularly in the Gamo and Wolaita zones, is constrained by seasonal feed shortages, high reliance on costly commercial concentrates, and underutilisation of locally available feed resources.

**Objectives:**

This study aimed to assess how socio‐economic and seasonal factors interact with the use of indigenous alternative feeds to enhance dairy productivity.

**Methods:**

A cross‐sectional survey was conducted involving 264 dairy‐producing households (132 per zone), selected through a multi‐stage sampling technique. Data were collected using structured questionnaires, focus group discussions, and field observations, and analysed using descriptive statistics, two‐way ANOVA, and index ranking.

**Results:**

The mean household size was 6.12 ± 2.16, and 62.2% of respondents were male. Commercial concentrate use was significantly higher in Gamo (61.6%) than in Wolaita (44.8%) (*p* < 0.001). Fresh/cut‐and‐carry feeding practices dominated in Gamo (82.9%), whereas Wolaita farmers preferred mixing alternative feeds with salt or grain (60.8%) (*p* < 0.001). Key indigenous species such as *Vernonia amygdalina* showed high preference indices (Gamo: 0.977; Wolaita: 0.985). Seasonal feed availability differed significantly between wet (30.8%) and dry seasons (20.5%) (*p* = 0.040). Improved animal health was more frequently reported in Wolaita (80.0%) than in Gamo (63.1%) (*p* = 0.002), while increased milk yield was reported by nearly all farmers (Gamo: 98.8%, Wolaita: 99.6%). Local availability and feeding value were the primary criteria for selecting indigenous fodder, with index values ranging from 0.689 to 0.803.

**Conclusions:**

The study concludes that indigenous feeds hold strong potential to address feed scarcity and enhance dairy productivity. Targeted extension, conservation techniques, and seed dissemination are recommended to strengthen adoption.

## Graphical abstract

The study assessed socio‐economic and seasonal factors in using indigenous alternative feeds such as *Vernonia amygdalina* that hold strong potential to address feed scarcity and enhance dairy productivity. Feed availability differed significantly between wet and dry seasons. Fresh feeding in Gamo and mixing with salt/grain in Wolaita were dominant practices with increased milk yield and improved animal health benefits frequently reported respectively. Local availability and feeding value were the primary criteria for selecting indigenous fodder.

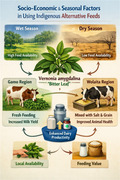

## 1 Introduction

Smallholder dairy farming constitutes a vital pillar of rural livelihoods in the tropics in general and southern Ethiopia in particular. The sector contributes significantly in provision of milk and milk products, income (approximately 50%) and employment to millions of households (Asrat et al. 2013; Gebre et al. 2023). About 80% of the population engaged in the sector (Shapiro et al. 2017). Despite its critical socio‐economic contributions, it is highly constrained by inadequate feed availability and suboptimal feed quality, both of which directly impede milk production and compromise animal health. Seasonal variability in forage availability is aggravated by increasing population pressure and land fragmentation. The situation further intensifies feed shortages, undermining the growth, productivity, and sustainability of smallholder dairy systems in the region (Tadesse et al. 2022). Therefore, addressing these feed‐related constraints is imperative to improving dairy performance and advancing rural development.

Indigenous feed resources, including native trees, shrubs, and herbaceous species, represent a largely underexploited yet promising complement to conventional feedstuffs in smallholder dairy production systems (Kibret et al. 2025). These species are well‐adapted in various administrative zones of southern Ethiopia and are inherently resilient to climatic fluctuations, such as prolonged dry spells and erratic rainfall patterns (Shigdaf et al. 2023). Many of these indigenous forages possess favourable nutritional attributes, including high protein, energy, and mineral content, making them particularly valuable during the dry season when the nutritional quality of natural pastures deteriorates (Atsbeha et al. 2020). The strategic use of such resources can reduce reliance on expensive commercial feeds, thereby improving both the economic viability and environmental sustainability of smallholder dairying.

Evidence suggests that supplemental feeding practices incorporating indigenous feed resources can significantly enhance milk yield, improve milk composition, and support reproductive efficiency and overall herd health in smallholder dairy systems (Balehegn et al. 2014). These benefits are, however, contingent on farmers’ awareness, access to planting and propagation materials, and prevailing market conditions (Derero and Kitaw 2018; Worku et al. 2022). Strengthening extension services, improving farmer training, and promoting community‐based forage production and conservation initiatives are essential to unlock the full potential of these alternative feed sources.

The integration of indigenous forages into smallholder dairy feeding regimes is increasingly recognised as a sustainable strategy to mitigate seasonal feed shortages while enhancing climate resilience and rural livelihoods (Terefe et al. 2023). The region (Gamo and Wolaita, the major contributors) is among the four regions with the greatest number of milking cows (Samuel et al. 2019) in the country, rich in a wide range of adaptable fodder species and has deep‐rooted experience of animal supplementation. Nevertheless, several constraints, such as limited availability of planting materials, land use competition, and inadequate agronomic knowledge, continue to hamper their widespread adoption (Nigussie et al. 2015). Overcoming these barriers requires targeted research, supportive policy frameworks, and participatory capacity‐building efforts.

Accordingly, this study seeks to investigate the socio‐economic characteristics, existing foliage feeding practices and perceived benefits associated with the use of indigenous supplemental feeds in the smallholder dairy systems of southern Ethiopia. By assessing farmers’ preferences, feed resource availability, and the nutritional contributions of indigenous species, the research aims to inform evidence‐based interventions that foster sustainable feed systems and improved dairy productivity. Ultimately, the effective utilisation of indigenous feed resources holds significant promise for enhancing food security, household incomes, and resilience to environmental and market‐related shocks among smallholder dairy farming communities.

## 2 Materials and Methods

### 2.1 Description of the Study Area

**FIGURE 1:**
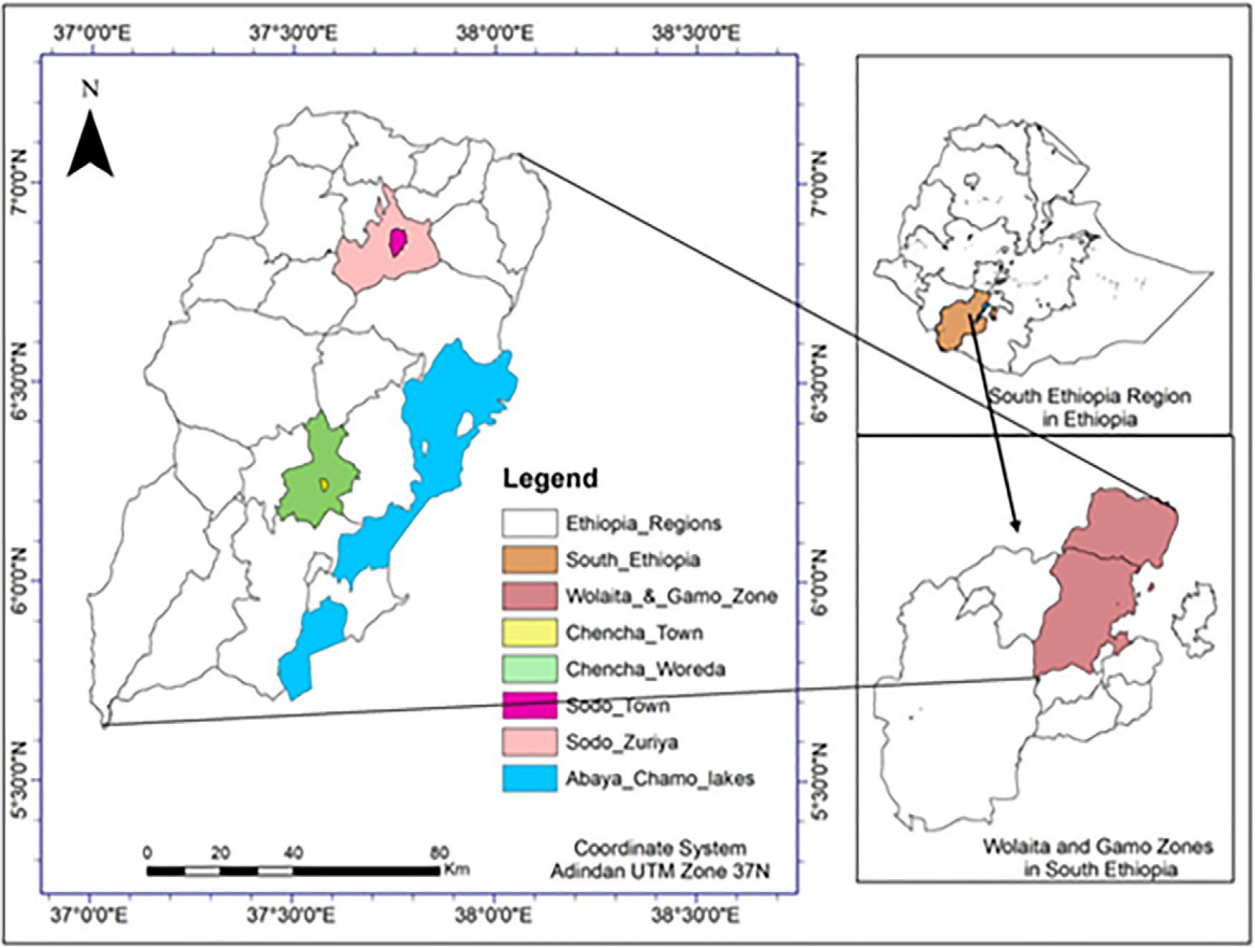
Enlarged administrative map of the study area.

The study was conducted in two administrative zones of south Ethiopia, namely, Gamo and Wolaita zones, respectively (Figure 1). The study districts, Chencha Zuria and Chencha town from Gamo and Sodo City and Sodo Zuria district from Wolaita zones, were selected based on dairy production potential and the wider availability of alternative feed resources and longer experiences used for cross‐bred dairy cattle. Chencha Zuria district is situated between 1300 and 3250 m above sea level. The astronomical location of Chencha district is between 37° 29′ 57′′ East and 37° 39′ 36′′ West, and between 60° 8′ 55′′ North and 60° 25′ 30″ South (CDLFRO 2020). Sodo Zuria district is situated between 1500 and 2750 m above sea level, and it has a 6°54 N, 37° 45 E latitude and 6.900°N, 37.750°E longitude astronomical location (WZFEDO 2017). The south Ethiopia is among the four regions with the greatest number of milking cows (Samuel et al. 2019) in the country and Gamo and Wolaita zones are known milk sheds in the region. The two seasons wet (June to September) and dry (October to January) are the main seasons of the study area according to Ethiopian Meteorological Agency.

### 2.2 Sampling Technique

A multi‐stage sampling procedure was employed to select dairy‐producing households from two administrative zones. In the first stage, two districts and two town administrations (one of each per zone) were purposefully selected based on dairy production potential among districts in the zone, population of crossbred dairy cattle, traditions of using alternative feed resources, and availability of such resources. In the second stage, three *kebeles* (local administrative units) were randomly selected from each potential district and each town, based on milk production potential and the experience of using alternative feed resources. In the final stage, a total of 264 dairy producers, 132 from each zone, were selected using a simple random sampling technique. All selected households owned at least one crossbred dairy cow and were actively using alternative feed resources to supplement the diet of milking cows. The total sample size was determined using the formula proposed by Yemane (1967) as follows:


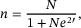

where *N* = study population, *n* = required sample size, *e* = expected per cent of error.

### 2.3 Data Collection

This study employed both primary and secondary data sources. Primary data were collected through household surveys, direct field observations, focus group discussions, and key informant interviews. Secondary data were obtained from a range of published and unpublished materials, including books, theses, technical reports, peer‐reviewed journals, government records, and statistical yearbooks.

A structured questionnaire, which was pre‐tested for validity and reliability, was used to gather detailed information on various aspects of dairy farming. These included the socio‐economic characteristics of respondents, dairy cattle breeds, feed resources, availability and utilisation of alternative feed resources, feeding practices, supplementation strategies for lactating cows, concentrate feeding practices and objectives, pricing and accessibility of concentrate feeds, processing methods of alternative feed resources, and perceived benefits of these practices.

The questionnaire was pilot‐tested in each district prior to the main data collection phase to refine the wording and structure. After revision based on the pilot test, the final version was administered by trained enumerators to ensure consistency and data quality.

### 2.4 Data Analysis

#### 2.4.1 Descriptive and Inferential Analysis

The collected data were analysed using the Statistical Package for Social Sciences (SPSS), version 27. Descriptive statistics were employed to summarise key variables. Statistical differences in categorical variables were assessed using cross‐tabulations, and significance was determined at *p* < 0.05. Quantitative data involving comparisons across locations and seasons were analysed using two‐way analysis of variance (ANOVA). When significant differences were observed, Tukey's Honestly Significant Difference (HSD) test was applied to separate the means. The following general linear model was used to analyse data related to identify feed resources and their utilisation practices:

$$Y_{\mathrm{ijk}}=\mu +\alpha_{i}+\beta_{j}+\alpha \beta_{\mathrm{ij}}+\sum_{\mathrm{ijk}},$$

where *Y_ijk_* = the total observation due to *i*, *j* and *k*; *μ* = the overall mean; *α_i_*_=_ the *i*th effect of location, *β_j_* the *j*th effect of seasonal availability of feed resources, *αβ_ij_*, the *ij*th interaction effect of location and season; *ε_ijk_* = the random error.

#### 2.4.2 Index Calculation for Ranking Parameters

To rank variables based on their relative importance, an index was calculated using the method described by Musa et al. (2006). The index was computed using the following formula:

$$\mathrm{Index}=\frac{\left(3\times \mathrm{Number}\;\mathrm{of}\;\mathrm{households}\;\mathrm{ranked}\;\mathrm{first}\right)+\left(2\times \mathrm{Number}\;\mathrm{of}\;\mathrm{households}\;\mathrm{ranked}\;\mathrm{second}\right)+\left(1\times \mathrm{Number}\;\mathrm{of}\;\mathrm{households}\;\mathrm{ranked}\;\mathrm{third}\right)}{\sum \left(3\times \mathrm{Number}\;\mathrm{of}\;\mathrm{households}\;\mathrm{ranked}\;\mathrm{first}\right)+\left(2\times \mathrm{Number}\;\mathrm{of}\;\mathrm{households}\;\mathrm{ranked}\;\mathrm{second}\right)+\left(1\times \mathrm{Number}\;\mathrm{of}\;\mathrm{households}\;\mathrm{ranked}\;\mathrm{third}\right)}.$$

This index was used to rank reasons, criteria, or preferences related to alternative feed resources. A higher index value indicated greater economic or practical importance as perceived by respondents. Ranking determinations were conducted based on two key dimensions: (1) priority based on availability and abundance of alternative feed resources and (2) priority based on perceived significance or utility. The indices were calculated separately for each criterion to facilitate comparative analysis.

## 3 Results

### 3.1 Comparative Analysis of Socio‐Demographic Variables Among Dairy Farmers

The sex distribution of respondents was fairly balanced across the two zones (Table 1). In the Gamo zone, 61.4% of respondents were male, while in the Wolaita zone, the figure was 62.9%, resulting in an overall average of 62.2% male and 37.9% female respondents. The difference between the zones was not statistically significant (*p* = 0.801).

**TABLE 1:** Socio‐demographic characteristics of smallholder dairy farmers in the studied area.

Variables	Locations (%)		Overall mean	*p* Value
	Gamo zone (*n* = 132)	Wolaita zone (*n* = 132)		
*Sex of respondents*				
Male	61.4	62.9	62.2	0.801
Female	38.6	37.1	37.9	
*Age of respondents*				
Less than 20 years	30	18.2	24.1	0.171
21–40 years	37.9	50.8	44.4	
More than 41 years	*40.2*	30.1	35.2	
*Educational status*				
Illiterate	*18.2*	12.9	15.6	0.191
Primary	54.5	54.5	54.5	
High school	4.6	3.9	4.3	
Certificate and above	22.7	28.9	25.8	
*Occupation of HH*				
Government worker	2.3	15.2	8.8	<0.001
Trader	4.5	15.2	9.9	
Retired people	1.5	5.3	3.4	
Farmer	91.7	64.4	78	
*Income source*				
Crop sale	59.9	52.3	56.1	0.308
Dairy products	31.1	37.9	34.5	
Salary	9.1	9.9	9.5	
Family size* *(year)*				
Less than 20 years	28	26.5	27.3	0.242
21–30 years	15.2	21.2	18.2	
31–40 years	10.6	11.4	11	
40–50 years	24.2	31.8	28	
More than 50 years	22	9.1	15.6	
*Marital status*				
Married	97	99.6	98.3	0.511
Single	2.2	0	1.1	
Widowed	0.8	0.8	0.8	

Abbreviations: HH, household head; *n*, number of respondents.

Age‐wise, 30% of Gamo zone respondents were under 20 years of age, compared to 18.2% in Wolaita. The 21‐40‐year group comprised 37.9% in Gamo and 50.8% in Wolaita, while those over 41 years represented 40.2% and 30.1% in Gamo and Wolaita zones, respectively. Despite some variation, the differences across age groups were not statistically significant (*p* = 0.171).

Primary education was the most common level attained in both zones, with 54.5% of respondents in each zone falling into this category. Illiteracy was more prevalent in Gamo (18.2%) than in Wolaita (12.9%), while higher education (certificate and above) was more common in Wolaita (28.9%) than in Gamo zone (22.7%). However, these differences were not statistically significant (*p* = 0.191). Significant differences were observed in household head occupations between the zones (*p* < 0.001). In Gamo, 91.7% of respondents were farmers compared to 64.4% in Wolaita. Wolaita zone had higher proportions of government workers (15.2%), traders (15.2%), and retired individuals (5.3%) compared to Gamo zone.

Crop sales were the dominant source of income for both Gamo (59.9%) and Wolaita (52.3%) zone respondents. Dairy products accounted for 31.1% of income in Gamo and 37.9% in Wolaita, while salaries contributed 9.1% and 9.9%, respectively. These differences were not statistically significant (*p* = 0.308).

The mean household size across both zones was 6.12 ± 2.16 persons. Distribution patterns showed that the majority of households fell in the 40–50 years category (28%), followed by those above 50 years (15.6%). There was no statistically significant difference between the zones (*p* = 0.242). Married respondents constituted the majority in zones, 97% in Gamo and 99.6% in Wolaita. Single and widowed individuals were very few, and differences across zones were statistically insignificant (*p* = 0.511).

### 3.2 Distribution and Utilisation of Supplementary and Basal Feeds in Smallholder Dairy Farming

The distribution of supplementary feeds used for milking cows across two administrative zones (Gamo and Wolaita) and two seasons (wet and dry) is summarised in Table 2. The most frequently used supplementary feed overall was commercial concentrate, with a significantly higher proportion in the Gamo zone (61.6%) compared with the Wolaita zone (44.8%) (*p* < 0.001). Seasonally, its use was greater during the dry season (29.9%) than during the wet season (22.3%) (*p* = 0.011). However, there was no significant interaction effect between location and season.

**TABLE 2:** Distribution of commonly used supplementary feeds for milking cows in the study area.

Variables	Location		Season		*p* Value		
Supplementary feed type	Gamo (%)	Wolaita (%)	Wet (%)	Dry (%)	Location (L)	Season (S)	L*S
Commercial concentrate	61.6	44.8	22.3	29.9	<0.001	0.011	NS
Alternative feeds (e.g., *V. amygdalina* and others)	22.8	25.1	12.5	11.4	0.70	<0.001	NS
Homemade concentrate	7.6	19.0	6.4	6.8	0.007	0.49	NS
Mix: concentrate + wheat bran + homemade + alternative feeds	3.8	8.4	3.4	3.8	0.14	0.30	NS
Wheat bran only	3.0	2.3	0.4	2.3	0.69	0.042	NS
Improved forages	1.5	0.8	—	1.1	0.56	0.32	NS

Abbreviations: L, location; L*S, location and season interaction; NS, non‐significant; S, season.

Alternative feeds such as *Vernonia amygdalina* were used at comparable rates in Gamo (22.8%) and Wolaita (25.1%) (*p* = 0.70). Their utilisation differed significantly between seasons (*p* < 0.001), being more common during the wet season (12.5%) than the dry season (11.4%).

Homemade concentrates were used significantly more in Wolaita (19.0%) than in Gamo (7.6%) (*p* = 0.007), with no notable seasonal variation (*p* = 0.49). Mixed feed formulations (combinations of commercial concentrates, wheat bran, homemade mixtures, and alternative feeds) were more frequently reported in Wolaita (8.4%) than in Gamo (3.8%), though this difference was not statistically significant (*p* = 0.14). Similarly, the seasonal variation for mixed feeds was not significant (*p* = 0.30).

Wheat bran alone was rarely used, showing no significant difference between locations (*p* = 0.69) but exhibiting a modest seasonal effect (*p* = 0.042), with higher use during the dry season. Improved forages were reported at low frequencies in both Gamo (1.5%) and Wolaita (0.8%), with no significant effect of location or season.

The principal basal feed resources identified in both study areas included natural pasture, crop residues, hay, enset by‐products (leaves, corms, and pseudo‐stems), avocado wastes, improved forages, commercial concentrates, and non‐conventional feeds. Substantial variation was observed in the type and utilisation patterns of these resources between zones and across seasons. Natural pasture was the predominant basal feed, reported by all respondents (100%), and followed by hay (62.5%). Crop residues were also widely used, with a significantly higher proportion in Wolaita (64.4%) than in Gamo (52.3%) (*p* = 0.033), particularly during the dry season (80.3%) (*p* < 0.001).

Enset by‐products represented a distinctive feed resource, being significantly more utilised in Gamo (78.0%) (*p* < 0.001) and primarily used during the dry season (*p* < 0.001). Avocado wastes, improved forages, and non‐conventional feeds (e.g. household leftovers and attela) were more frequently used in Wolaita (64.4%, 62.1%, and 50.8%, respectively) compared with Gamo (22.0%, 43.9%, and 49.2%, respectively). These feed resources also demonstrated significant seasonal variation (*p* = 0.024), with greater utilisation during the dry season.

### 3.3 Patterns in Supplementary Feed Acquisition: Insights From Household‐Level Data

**FIGURE 2:**
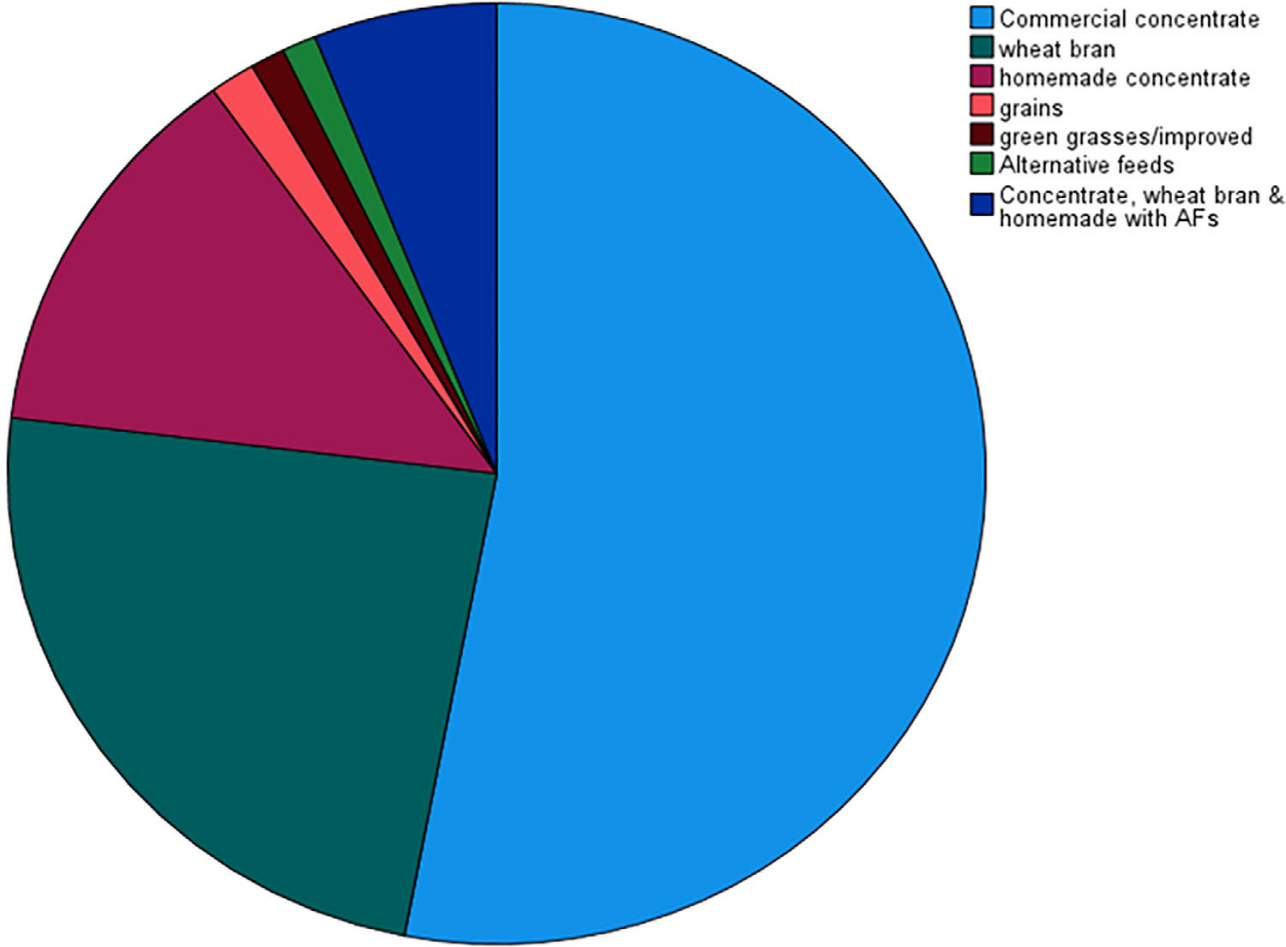
Sources from which common supplementary feeds are obtained in the study area.

The analysis of sources from which supplemental feeds are obtained by smallholder farmers reveals a diverse pattern of feed acquisition (Figure 2). The majority of respondents (47%) reported sourcing supplemental feeds primarily from their own land. Approximately 28% of farmers obtain supplemental feeds through farmers' unions. Feeds purchased from local markets accounted for 17% of the sources. A smaller portion of respondents (6%) accessed supplemental feeds from individual distributors. Only 2% of farmers reported obtaining supplemental feeds from everywhere.

### 3.4 Alternative Feed Resources for Supplementing Milking Cows in the Study Areas

The study identified diverse alternative supplemental feed resources for milking cows in the Gamo and Wolaita zones, with notable variation in species richness and preference rankings between the two areas (Table 3). In total, 12 species were ranked and indexed by respondents in the Gamo zone, while 33 species were ranked in Wolaita, suggesting broader recognition or availability of alternative feeds in Wolaita.

**TABLE 3:** Alternative supplemental feeds for milking cows identified in the study areas.

		Locations (administrative zones)			
Identified alternative feed resources		Gamo (*n* = 132)		Wolaita (*n* = 132)	
Local name	Scientific name	Index	Rank	Index	Rank
Garaa	*Vernonia amygdalina*	0.977	1	0.985	1
Itriwanjiyaa	*Ehretia cymosa*	—	—	0.648	2
Halilo	*Hovenia dulcis*	0.580	2	—	—
Okaa	*Cirsium vulgare*	0.538	3	—	—
Haytta Tukiyaa	*Coffee arabica*	—	—	0.500	3
Uutta	*Ensete ventricosum*	0.477	4	—	—
Lolashee	*Dombeya torrida*	0.470	5	—	—
Danbursa	*Pentas schimperiana*	—	—	0.462	4
Higisha Meruwaa	*Clutia lanceolata*	—	—	0.458	5
Guluwaa	*Ocimum gratissmum*	—	—	0.447	6
Shaashaa	*Lippia adoensis *	—	—	0.417	7
Boorisaa	*Echinops kebericho*	0.348	8	0.470	8
Kirkisaa	*Leucas calostachys*	—	—	0.398	9
Woyshaa wurcee	*Arundinaria alpina*	0.379	6	—	—
Kaffo wosolluwaa	*Impatiens hochstetteri*	—	—	0.337	10
Alaga Buluwaa	*Solanum incanum*		—	0.330	11
Kosso mitta	*Hagenia abyssinica*	0.379	6	0.257	12
Muldhaa	*Plectranthus caninus*	0.311	9	0.311	13
Cacuwaa	*Premna schimperi*	—	—	0.280	14
Tulluwaa	*Helichrysum odoratissimum*	—	—	0.269	15
Bawiyaa	*Englerina woodfordioides*	—	—	0.258	16
Bortuwaa hayttaa	*Erythrina abyssinica*	0.220	10	—	—
Dormiyaa	*Guizotia abyssinica*	—	—	0.208	17
Modooruwaa	*Funastrum clausum*	—	—	0.197	18
Aydaamiyaa	*Acmella caulirhiza*	0.170	11	0.170	19
Dadaa turaa	*Dolichandra unguis*	—	—	0.170	20
Daldhishaa	*Commelina benghalensis*	0.140	12	0.140	21
Saasiyaa	*Erica arborea*	—	—	0.110	22
Zeldhumaa	*Rumex nepalensis*	—	—	0.080	23
Gumariyaa	*Grewia mollis*	—	—	0.076	24
Yiishinchaa	*Acokanthera schimperi*	—	—	0.072	25
Macca qirqaa	*Hypericum grandifolium*	—	—	0.068	26
Shaakishuwaa	*Myrsine guianensis*	—	—	0.061	27
Qanqarsaa	*Myrsine africana*	—	—	0.053	28
Ximbaala	*Jasminum floribundum*	—	—	0.045	29
Gergecuwaa	*Maesa lanceolata*	—	—	0.042	30
Micakuwaa	*Rhus natalensis*	—	—	0.030	31
Meegaraa	*Euclea schimperi*	—	—	0.027	32
Shomoluwa	*Olinia rochetiana*	—	—	0.019	33

Abbreviation: *n*, number of respondents.

In both administrative zones, *Vernonia amygdalina* (*Garaa*) was unanimously identified as the top‐ranked species with the highest index values, 0.977 in Gamo and 0.985 in Wolaita indicating its perceived importance and wide acceptance as a supplemental feed. In Gamo, *Hovenia dulcis* (*Halilo*), *Cirsium vulgare* (*Okaa*), *Ensete ventricosum* (*Uutta*), and *Dombeya torrida* (*Lolashee*) followed in the top five rankings with index values ranging from 0.580 to 0.470. In Wolaita, *Ehretia cymosa* (*Itriwanjiyaa*), *Coffee arabica* (*Haytta Tukiyaa*), *Pentas schimperiana* (*Danbursa*), and *Clutia lanceolata* (*Higisha Meruwaa*) were ranked in positions 2 to 5 with index values between 0.648 and 0.458.

Some species such as *Echinops kebericho, Plectranthus caninus*, and *Hagenia abyssinica* were shared between both zones but differed in ranks and index values. A large number of species (21) were uniquely identified and ranked only in Wolaita, including lower‐indexed species like *Olinia rochetiana* (*Shomoluwa*) and *Euclea schimperi* (*Meegaraa*), indicating broader local ethnobotanical knowledge, longstanding experience of supplementing milking cows with these feed resources or forage biodiversity in that area.

### 3.5 Criteria Influencing the Selection of Indigenous Fodder Species Among Smallholder Farmers

The ranking and index scores of criteria used by farmers in Gamo and Wolaita zones for selecting preferred indigenous fodder species are summarised in Table 4. Across both locations, local availability emerged as the most important criterion, with the highest index values in Gamo (0.758) and Wolaita (0.803), and the top rank (1) in both zones. Seasonally, local availability was also the dominant factor, more emphasised during the dry season (45.6%) compared to the wet season (32.3%).

**TABLE 4:** Criteria for selecting preferred indigenous herb/fodder species.

Selection criteria	Locations (zones)					
	Gamo (*n* = 132)		Wolaita (*n* = 132)		Season	
	Index	Rank	Index	Rank	Dry %	Wet %
Local availability	0.758	1	0.803	1	45.6	32.3
Feeding value	0.719	2	0.689	2	36.9	33.4
Multipurpose use	0.462	3	0.545	3	25.1	25.5
Cost/for free	0.530	4	0.455	4	26.6	22.8
Medicinal value	0.379	5	0.447	5	20.9	20.5
Biomass yield	0.235	6	0.220	6	15.2	7.6
Palatability	0.205	7	0.152	7	7.6	10.7

Abbreviation: *n*, number of respondents.

Feeding value was the second most important criterion in both zones, with indices of 0.719 (Gamo) and 0.689 (Wolaita), and ranked second in both areas. It was similarly emphasised across seasons (36.9% dry, 33.4% wet). Multipurpose use (e.g., for food, construction, or cultural roles) ranked third in both zones, with comparable importance in both seasons (25.1% in dry vs. 25.5% in wet).

Cost or free availability and medicinal value followed in fourth and fifth place, respectively. While cost was slightly more important in Gamo (index = 0.530) than in Wolaita (0.455), medicinal value was marginally more valued in Wolaita. Both criteria showed modest seasonal variation.

Biomass yield and palatability were the least prioritised criteria, ranked sixth and seventh in both zones. Biomass yield was more considered during the dry season (15.2%) than in the wet (7.6%), whereas palatability had slightly higher emphasis during the wet season (10.7%) than the dry (7.6%).

### 3.6 Spatial and Seasonal Variation in the Availability of Alternative Supplemental Feeds

The spatial and seasonal availability of alternative supplemental feeds for dairy cattle in Gamo and Wolaita zones are presented in Table 5. The ‘relatively available’ category was most commonly reported in both zones, cited by 51.7% of respondents in Gamo and 50.9% in Wolaita. No significant difference was observed between the two locations (*p* = 0.727). However, a significant seasonal difference was evident (*p* = 0.040), with higher availability reported during the wet season (30.8%) compared to the dry season (20.5%). Furthermore, the interaction between location and season was significant (*p* = 0.001), suggesting that seasonal changes affect each zone differently.

**TABLE 5:** Availability of alternative supplemental feeds in the study area.

	Locations (%, zones)		Season (%)		*p* Value		
Availability category	Gamo (*n* = 132)	Wolaita (*n* = 132)	Wet	Dry	Location (L)	Season (S)	L*S
Relatively available	51.7	50.9	30.8	20.5	0.727	0.04	0.001
Sparsely available	30.4	34.2	20.9	11.4	0.48	0.022	NS
Poorly available	7.6	11.4	3.8	5.7	0.28	0.47	NS
Highly available	1.5	1.5	1.5	—	1.0	0.25	NS

Abbreviations: L, location; S, season; L*S, location and season interaction; *n*, number of respondents; NS, non‐significant.

Feeds that were sparsely available were more frequently reported in Wolaita (34.2%) than in Gamo (30.4%), although the difference was not statistically significant (*p* = 0.48). Seasonally, sparse availability was significantly higher in the wet season (20.9%) than in the dry season (11.4%) (*p* = 0.022), but there was no significant interaction between location and season.

The category ‘poorly available’ was reported by 7.6% of Gamo and 11.4% of Wolaita respondents. No significant differences were found across locations (*p* = 0.28) or seasons (*p* = 0.47). Lastly, high availability of alternative supplemental feeds was rare in both locations (1.5%) and only reported during the wet season, with no significant variation by location or season.

### 3.7 Seasonal Utilisation and Plant Part Preference of Alternative Feed Resources for Milking Cows

The analysis of alternative feed resources revealed a variety of plant species utilised for supplementing milking cows in the Gamo and Wolaita zones, differentiated by plant parts used and seasonal availability (Table 6). A total of 40 different species were identified. Leaves were the most commonly used plant part, followed by leaf + stem combinations, whole plants, roots, and outgrowths. Several species such as *Vernonia amygdalina*, *Ehretia cymosa*, *Coffee arabica*, and *Ocimum gratissimum* were reported to be used consistently across both zones and seasons, with 100% usage.

**TABLE 6:** Seasonal availability and plant part utilisation of alternative indigenous feed resources in Gamo and Wolaita zones, southern Ethiopia.

Identified alternative feed resources	Plant part used	Wet season (%)		Dry season (%)		Both seasons (%)		Overall mean	*p* Value
		Gamo	Wolaita	Gamo	Wolaita	Gamo	Wolaita		
*Vernonia amygdalina*	Leaf					100	100	100	0.814
*Ehretia cymosa*	Leaf						100	100	
*Hovenia dulcis*	Leaf + stem	95.4				4.6		50	
*Cirsium vulgare*	Leaf + stem	90.9				9.1		50	
*Coffee arabica *	Leaf						100	100	
*Ensete ventricosum*	Whole plant					100		100	
*Dombeya torrida*	Leaf					100		100	
*Pentas schimperiana*	Leaf + stem						100	100	
*Clutia lanceolata*	Leaf		15.2				84.8	50	
*Ocimum gratissmum*	Leaf						100	100	
*Lippia adoensis *	Leaf		15.2				84.8	50	
*Echinops kebericho *	Root	17	18.6	11.0	9.5	71.2	72.7	33.3	<0.001
*Leucas calostachys*	Leaf		5.3		3.8		90.9	33.3	
*Arundinaria alpina*	Leaf	5.3				94.7		50	
*Impatiens hochstetteri*	Leaf + stem		19.7				80.3	50	
*Solanum incanum*	Flower		15.2				84.8	50	
*Hagenia abyssinica*	Leaf + fruit	11	9.4			87.5	92.1	50	0.008
*Plectranthus caninus*	Leaf	27.6	26.1			72.4	73.9	50	0.329
*Premna schimperi*	Leaf		15.2				84.8	50	
*Helichrysum odoratissimum*	Leaf		15.2				84.8	50	
*Englerina woodfordioides*	Outgrowth		6.8		83.3		9.8	33.3	
*Erythrina abyssinica*	Leaf	5.3				94.7		50	
*Guizotia abyssinica*	Leaf		9.8		16.7		73.5	33.3	
*Funastrum clausum*	Leaf		13.6				86.4	50	
*Acmella caulirhiza*	Whole plant	71.6	73.9			28.4	26.1	50	0.069
*Dolichandra unguis‐cati*	Leaf		68.9				31.1	50	
*Commelina benghalensis*	Leaf + stem	76.9	73.9			23.1	26.1	50	0.069
*Erica arborea*	Leaf		1.5				90.9	46.2	
*Rumex nepalensis*	Leaf + stem		97				3	50	
*Grewia mollis *	Leaf		90.9				9.1	50	
*Acokanthera schimperi*	Leaf				90.9		9.1	50	
*Hypericum grandifolium*	Leaf		1.5				98.5	50	
*Myrsine guianensis*	Leaf		3.8				94.7	49.3	
*Myrsine africana*	Leaf		2.3				97.3	50	
*Jasminum floribundum*	Leaf		1.5				98.5	50	
*Maesa lanceolata*	Leaf		9.1				89.4	49.3	
*Rhus natalensis*	Leaf		1.5				98.5	50	
*Euclea schimperi*	Leaf		7.6				90.9	49.3	
*Olinia rochetiana*	Leaf + stem		97				3	50	

Among species with significant seasonal variability, *Echinops kebericho* showed a highly significant difference in usage (*p* < 0.001), with greater utilisation during both seasons (71.2% and 72.7% in Gamo and Wolaita, respectively) compared to the wet and dry seasons separately. *Hagenia abyssinica* also demonstrated a significant seasonal variation (*p* = 0.008), being primarily used in both seasons rather than exclusively in wet or dry conditions.

Species like *Acmella caulirhiza* and *Commelina benghalensis* showed relatively high usage in the wet season (71.6%–76.9%) with a noticeable decline in the dry season, but without statistically significant differences (*p* > 0.05). Most of the remaining species, such as *Guizotia abyssinica*, *Rumex nepalensis*, and *Plectranthus caninus*, were predominantly used during both seasons, accounting for more than 70% usage.

Overall, the mean usage of alternative feed resources across both zones and seasons ranged from 33.3% to 100%, with the highest consistency observed in species used during both seasons. Only a few species demonstrated significant statistical differences (*p* < 0.05) in their seasonal utilisation patterns.

### 3.8 Feeding Practices and Benefits of Alternative Supplemental Feeds in Milking Cows

The feeding practices of alternative feeds (AFs) showed significant variation (*p* < 0.05) between the two zones, Gamo and Wolaita (Table 7). The feeding practices of alternative supplemental feeds (AFs) varied significantly between the Gamo and Wolaita zones and across seasons (dry and wet). In Gamo, feeding fresh or cut‐and‐carry AFs was the dominant practice, with 82.9% of respondents reporting this method. In contrast, this practice was not reported in Wolaita (0%). Seasonal variation was also significant: 61.6% of respondents used fresh/cut‐and‐carry feeding during the wet season compared to only 21.3% in the dry season (*p* < 0.001). Location and season, as well as their interaction (L*S), significantly affected this practice (*p* < 0.001, *p* = 0.002, and *p* < 0.001, respectively).

**TABLE 7:** Feeding practices of alternative supplemental feeds in the study areas.

	Locations (%, zones)		Season (%)		*p* Value		
Feeding practice of AFs	Gamo (*n* = 132)	Wolaita (*n* = 132)	Dry	Wet	Location (L)	Season (S)	L*S
Feeding as fresh/cut‐and‐carry	82.9	—	21.3	61.6	<0.001	0.002	<0.001
Feeding wilted AFs (*Cirsium vulgare*)	17.5	—	3.8	13.7	<0.001	0.003	—
Pounding and mixing with salt, grains, cottonseed, wastes	—	35.7	15.2	20.5	<0.001	0.004	—
Mixing pounded AFs with salt/salt bar	—	60.8	30.4	30.4	<0.001	—	—
Chopping and mixing with fresh grasses	—	3.8	2.3	1.5			

Abbreviations: AFs, alternative supplemental feeds; L, location; L*S, location and season interaction; *n*, number of respondents; S, season.

Feeding wilted AFs, specifically *Cirsium vulgare*, was reported only in Gamo (17.5%) and was significantly higher during the wet season (13.7%) than the dry season (3.8%) (*p* < 0.001). Pounding and mixing AFs with salt, grains, cottonseed, and agricultural wastes were exclusively reported in Wolaita (35.7%), with significant seasonal variation (15.2% dry vs. 20.5% wet; *p* < 0.001). Similarly, mixing pounded AFs with salt or salt bars was also unique to Wolaita (60.8%), with no significant seasonal variation noted.

Chopping and mixing AFs with fresh grasses was the least common practice, reported only in Wolaita (3.8%), with negligible seasonal differences.

### 3.9 Impact of Alternative Feed Resources on Milk Yield, Quality, and Health of Milking Cows

The findings presented in Table 8 reveal that farmers in both Gamo and Wolaita zones overwhelmingly recognised the benefits of alternative supplemental feeds (AFs) for milking cows. A particularly high proportion of respondents reported an increase in milk yield, with 98.8% in Gamo and 99.6% in Wolaita acknowledging this benefit. Seasonal variation was minimal, with 49.4% in the wet season and 49.9% in the dry season reporting improved milk yield. There were no statistically significant differences observed between zones, seasons, or their interaction for this indicator (*p* > 0.05).

**TABLE 8:** Benefits of alternative supplemental feeds in milking cows.

	Locations (%, zones)		Season (%)		*p* Value		
Feeding benefits of AFs	Gamo (*n* = 132)	Wolaita (*n* = 132)	Wet	Dry	L	S	L*S
Increased milk yield	98.8	99.6	49.4	49.9	1.0	1.0	1.0
Improved body condition	73.7	76.8	37.2	38	0.670	1.0	0.891
Improved milk composition/butter yield	76	80.6	39.5	38.8	0.457	1.0	0.894
Improved milk appearance	85.1	86.6	43.7	42.2	0.861	0.901	1.0
Provision of essential nutrients	71.4	70	35.7	35	0.893	1.0	1.0
Improved animal health	63.1	80	35.3	36.1	0.002	1.0	0.324

Abbreviations: AFs, alternative supplemental feeds; L, location; L*S, location and season interaction; *n*, number of respondents; S, season.

Similarly, improvement in body condition of milking cows due to AFs was reported by 73.7% and 76.8% of respondents in Gamo and Wolaita, respectively. Seasonally, 37.2% in the wet season and 38.0% in the dry season perceived this benefit. However, the statistical analysis showed no significant difference across locations (*p* = 0.670), seasons (*p* = 1.0), or their interaction (*p* = 0.891).

Regarding milk composition and butter yield, 76.0% of Gamo and 80.6% of Wolaita respondents reported improvements. Seasonal trends remained stable (wet: 39.5%; dry: 38.8%), and no significant differences were detected across any of the tested factors (*p* > 0.05).

Milk appearance was noted as improved by 85.1% in Gamo and 86.6% in Wolaita. Responses across seasons were consistent, with 43.7% in the wet and 42.2% in the dry season acknowledging this benefit. No statistically significant variations were recorded by location (*p* = 0.861), season (*p* = 0.901), or their interaction (*p* = 1.0).

The provision of essential nutrients was cited by 71.4% in Gamo and 70.0% in Wolaita. The seasonal distribution was nearly identical (wet: 35.7%, dry: 35.0%), and statistical tests showed no significant differences across location, season, or their interaction (*p* > 0.05).

Interestingly, improved animal health was reported by a significantly higher proportion of farmers in Wolaita (80.0%) compared to Gamo (63.1%). Although seasonal responses were similar (wet: 35.3%; dry: 36.1%), a statistically significant difference was observed between locations (*p* = 0.002), indicating a location‐specific perception or experience regarding this benefit. However, no significant seasonal effect or interaction was observed (*p* = 1.0 and *p* = 0.324, respectively).

## 4 Discussion

### 4.1 Socio‐Economic Profiles and Livelihood Dynamics of Smallholder Dairy Farmers: Implications for Rural Development Strategies

The socio‐economic characteristics of respondents suggest a fairly uniform demographic profile between the Gamo and Wolaita zones, with the exception of occupational status (Table 1). The male dominance in survey responses is consistent with findings from other rural Ethiopian contexts, where men are more likely to be designated as household heads and more frequently participate in formal surveys (Gebremariam et al. 2021).

The age distribution implies that the agricultural workforce is relatively young to middle‐aged, particularly in Wolaita. This demographic structure may be beneficial for labour‐intensive farming practices and the adoption of agricultural innovations, as younger farmers are often more receptive to new technologies (Belay et al. 2022).

Educational attainment, though moderate, shows encouraging trends with over half of the respondents having completed primary education. This can significantly impact the uptake of extension services and improved farming techniques. Similar patterns have been reported in other parts of southern Ethiopia (Tadesse et al. 2020), emphasising the importance of education in rural transformation.

The significant differences in occupation highlight greater livelihood diversification in the Wolaita zone, possibly due to better access to non‐farm employment opportunities. This aligns with recent studies indicating increased urbanisation and informal sector participation in Wolaita (Yilma and Hailu 2023). Conversely, the Gamo zone remains predominantly agrarian, which may limit income resilience during agricultural shocks.

The dominance of crop and dairy sales as income sources reflects the agrarian economy's dependence on both crop cultivation and livestock production. However, the relatively equal distribution between the two suggests that dairy production could be a strategic area for livelihood improvement in both regions, particularly for women, who often engage in milk marketing (FAO 2022).

Family size findings are in line with national averages and have implications for household labour availability. Larger families can provide more labour for agricultural tasks, although they also imply higher consumption needs, which can strain limited resources (CSA 2021). Marital stability, as shown by the high percentage of married respondents, typically enhances household cooperation in agricultural activities and long‐term planning.

### 4.2 Seasonal and Spatial Variation in Supplementary Feed Use Among Smallholder Dairy Farmers

The results demonstrate considerable variation in the use of supplementary feeds for milking cows across locations and seasons in the study area. The significantly higher usage of commercial concentrates in Gamo zone compared to Wolaita may reflect differences in socioeconomic status, access to markets, and extension service delivery. Farmers in Gamo might have better purchasing power or proximity to urban centres where feed outlets and veterinary services are more available. Similar regional disparities in concentrate feed use have been reported in other parts of southern Ethiopia, often driven by infrastructure, access to credit, and market orientation (Alemayehu et al. 2021; Mekonnen et al. 2023).

Seasonal effects were particularly notable for commercial concentrates and alternative feeds. The increased use of commercial feeds during the dry season is consistent with the expected forage scarcity that typically characterises this period. Farmers may resort to concentrates to maintain milk yield and animal condition, especially when natural pastures are depleted (Haileselassie et al. 2020). Likewise, the greater reliance on alternative feeds such as *Vernonia amygdalina* during the wet season may be attributed to their seasonal abundance and accessibility, as observed in various administrative zones of southern Ethiopia (Tesfaye and Tolera 2022).

Wolaita farmers' greater use of homemade concentrates suggests localised feed innovations or a greater reliance on cost‐effective alternatives to commercial feeds. This aligns with findings by Tadesse et al. (2023), who observed that homemade feed formulations are common in areas with limited market integration or among farmers with traditional knowledge of feed mixing. The absence of seasonal variation in the use of homemade feeds further suggests a stable year‐round feeding strategy, possibly supported by stockpiling or reliance on crop residues and agro‐industrial by‐products.

The low adoption of commercially mixed feeds and improved forages across both locations is concerning. Mixed rations are known to improve nutrient balance and enhance milk production efficiency, yet their use remains limited, possibly due to knowledge gaps or the absence of consistent extension follow‐up (Yami et al. 2022) and skyrocketed price of mixed rations. Similarly, the poor use of improved forages indicates under exploitation of technologies that could reduce seasonal feed gaps and enhance dairy productivity. Limited seed availability, land constraints, and inadequate farmer training are among the barriers frequently cited in similar contexts (FAO 2024; Gebremedhin et al. 2021). The situation encouraged expanded use of indigenous feed resources to exploit their advantages in terms of adaptability to the local environment, availability, and familiarity to the farmers (Asmare and Mekuriaw 2019).

In summary, the findings underscore the importance of tailored interventions that address location‐specific constraints while promoting seasonally adaptive feeding strategies. Strengthening extension services, improving input supply chains, and promoting forage development programs are critical to improving feed resource utilisation and dairy productivity in the region.

### 4.3 Sources and Strategies of Supplemental Feed Acquisition Among Smallholder Dairy Farmers

The results indicate a strong reliance on on‐farm feed production, reflecting farmers' efforts to utilise locally available resources and maintain sustainability in feed supply (Mekonnen et al. 2018). The significant role of farmers’ unions (28%) highlights the importance of collective organisation and cooperative purchasing in improving access to quality feed, which can enhance bargaining power and reduce costs for smallholder farmers (Gebremedhin and Jaleta 2010).

The contribution of local markets (17%) suggests that while they serve as an important feed source, their availability may be seasonal or geographically limited, affecting reliability (Kassie et al. 2011). The smaller share of individual distributors (6%) likely represents more specialised or commercial suppliers offering specific feed types, but potentially at higher costs (Teshome et al. 2016).

The minimal percentage (2%) of farmers sourcing from mixed or opportunistic channels reflects strategies used during feed scarcity (Alemayehu et al. 2014). Overall, the findings underscore the need to promote improved on‐farm feed production alongside strengthening farmers’ unions and market linkages to enhance feed availability, reduce costs, and support sustainable dairy productivity in southern Ethiopia (Teklewold et al. 2013).

### 4.4 Criteria Influencing the Selection of Indigenous Fodder Species Among Smallholder Farmers

The selection of indigenous fodder species by farmers in Gamo and Wolaita zones (Table 4) reflects a strong preference for practical and immediate accessibility, with local availability and feeding value topping the criteria list. This underscores the resource‐constrained nature of smallholder systems, where farmers rely heavily on nearby vegetation and traditional foraging routes to meet livestock nutritional needs (Mekonnen et al. 2023; Yisehak et al. 2021). The higher emphasis on local availability during the dry season is consistent with the seasonal scarcity of herbaceous forage, compelling farmers to depend more on accessible indigenous species (Tesfaye and Tolera 2022).

Feeding value's consistent ranking in both zones and seasons reveals farmers’ keen observational knowledge of animal performance in relation to specific herbs. This traditional experiential understanding of which species promote better body condition, milk yield, or health has been documented widely across East Africa (Alemayehu et al. 2021). However, the relatively modest importance placed on biomass yield and palatability suggests that farmers prioritise feed availability over nutritional optimisation, likely due to land limitations and the need to ensure consistent roughage supply.

The recognition of multipurpose uses of fodder species, such as for firewood, soil fertility, or medicinal purposes, highlights the integrated nature of smallholder farming systems. Species that fulfil both livestock and household needs are more likely to be protected and propagated (FAO 2024). This multipurpose criterion also reflects gendered roles in fodder management, where women often prioritise species with utility beyond livestock feeding (Gebremedhin et al. 2022).

The relatively high rank of cost or free access further affirms the economic vulnerability of these systems. Farmers in both zones prefer species that do not require purchase, transportation, or labour‐intensive harvesting, emphasising the importance of community‐shared grazing lands and roadside foraging (Tadesse et al. 2023). The medicinal value of certain herbs for both humans and livestock, while less prioritised, remains an important criterion in traditional systems, especially where veterinary services are limited.

In contrast, the low ranking of palatability suggests that while taste and voluntary intake are important, they are secondary to availability and multipurpose function. This may reflect a compromise strategy: farmers feed what is available, regardless of preference, particularly during feed‐scarce periods. This behavioural trade‐off aligns with recent findings in feed‐scarce highland zones of Ethiopia (Yami et al. 2022).

Overall, this study highlights the intersection of ecological, economic, and socio‐cultural factors shaping indigenous fodder use. For sustainable forage development, extension strategies should prioritise locally abundant species with high nutritive value and multiple uses, while promoting community‐based selection and conservation programs.

### 4.5 Seasonal Dynamics and Constraints of Alternative Supplemental Feed Resources

The availability of alternative supplemental feeds, such as indigenous shrubs, leaves of multipurpose trees or agro‐industrial by‐products, plays a crucial role in maintaining livestock productivity, especially in feed‐scarce regions. The predominance of the ‘relatively available’ category in both Gamo and Wolaita suggests that while these resources are present, their supply is moderate and not consistently dependable, particularly during the dry season (Haileselassie et al. 2020; Mekonnen et al. 2023).

The significant seasonal variation in the availability of alternative feeds, especially under the ‘relatively available’ and ‘sparsely available’ categories, highlights the cyclical nature of feed resources. The wet season promotes vegetative growth, increasing the availability of herbaceous species and browsable plants like *Vernonia amygdalina*, *Sesbania sesban*, and others (Tesfaye and Tolera 2022). In contrast, during the dry season, many of these species either lose their leaves or are overharvested, exacerbating feed shortages.

The statistically significant location‐by‐season interaction effect (*p* = 0.001) for relative availability suggests that farmers in the two zones are affected differently by seasonal patterns. This may be due to variations in agroecological conditions, land use patterns, and community resource management strategies. For instance, some areas may have communal forest patches or grazing reserves that buffer dry‐season shortages, while others may lack such infrastructure (Alemayehu et al. 2021; FAO 2024).

The low incidence of ‘highly available’ responses in both zones, and only during the wet season, confirms the limited surplus nature of alternative supplemental feeds. This scarcity poses a challenge to sustainable livestock feeding, especially for dairy cows with high nutritional demands. It suggests a lack of systematic forage conservation, such as hay‐making, silage production, or fodder tree plantation, which could otherwise improve feed availability during the lean seasons (Gebremedhin et al. 2021).

The findings also reflect the limited integration of alternative feeds into formal feeding systems, indicating a need for extension programs to support smallholders in identifying, conserving, and sustainably managing these resources. Increasing awareness and capacity around feed preservation techniques, local feed resource mapping, and forage establishment would help stabilise feed availability and improve dairy performance throughout the year (Yami et al. 2022).

### 4.6 Seasonal Dynamics and Plant Part Utilisation of Alternative Feed Resources in Smallholder Dairy Production Systems

The observed diversity of alternative feed resources reflects the adaptive strategies employed by smallholder farmers to mitigate seasonal feed shortages for milking cows in the Gamo and Wolaita zones. The predominance of leaves as the preferred plant part followed by stems, whole plant, fruits/seeds, root and flowers. This finding aligns with recent findings emphasising foliage as a highly palatable and nutrient‐rich component suitable for ruminant diets in tropical systems (Lemma et al. 2023; Negash et al. 2023). This preference also supports sustainable feeding practices by allowing regrowth of plants, thus maintaining forage availability over time (Abera et al. 2022).

Consistent year‐round use of species such as *Vernonia amygdalina* and *Ocimum gratissimum* indicates their importance as reliable feed sources, corroborating studies highlighting their nutritive value and resilience across locations (Bekele et al. 2022; Dida et al. 2021). These species’ adaptability makes them central to feed security strategies in mixed crop‐livestock systems, particularly under fluctuating climatic conditions.

Seasonal variability in the use of species like *Echinops kebericho* and *Hagenia abyssinica* underscores the influence of plant phenology and environmental factors on feed resource availability. Similar observations have been reported where multipurpose indigenous shrubs and trees provide critical feed during dry seasons but show limited availability during wet periods (Abera et al. 2022; Teshome et al. 2021). This seasonal pattern necessitates integrated feed management to buffer against feed scarcity and sustain milk production.

The wet‐season preference for species such as *Acmella caulirhiza* and *Commelina benghalensis*, though not statistically significant, reflects their growth cycles and moisture dependency. Conversely, species utilised predominantly in the dry season may serve as key resources when fresh forage is limited, highlighting the need for conserving drought‐resilient species to enhance system resilience (Bekele et al. 2022; Lemma et al. 2023).

Overall, the high consistency in using several species across both seasons suggests the existence of a core group of indigenous plants that support livestock nutrition year‐round. Promoting the sustainable harvesting, propagation, and incorporation of these species into fodder production systems can enhance smallholder dairy productivity and resilience in the face of climate variability (Negash et al. 2023; Teshome et al. 2021).

### 4.7 Seasonal and Locational Variation in Feeding Practices of Alternative Supplemental Feeds for Dairy Cows

The distinct feeding practices between the two administrative zones reflect variations in local livestock management systems, cultural preferences, and availability of feed resources (Table 7). The predominance of fresh or cut‐and‐carry feeding of the alternative supplemental feeds in Gamo aligns with smallholder practices that emphasise direct utilisation of green fodder, especially during the wet season when forage availability is higher (Lemma et al. 2023; Negash et al. 2023). This method ensures minimal processing and rapid feed delivery, which may enhance nutrient intake and animal performance.

Conversely, the Wolaita zone's preference for pounding and mixing of the AFs with salt, grains, cottonseed, or wastes for stall‐feeding/indoor feeding is a unique practice and suggests a more processed and supplemented feeding strategy which is not common in Gamo case. This could be driven by the need to improve feed palatability, nutrient density, or storage convenience, especially in a system where feed scarcity is more pronounced during dry periods (Bekele et al. 2022; Teshome et al. 2021). The addition of salt or salt bars is consistent with known mineral supplementation practices that support animal health and productivity.

Feeding wilted AFs like *Cirsium vulgare* in Gamo primarily during the wet season likely reflects local knowledge on improving feed palatability and reducing anti‐nutritional factors through wilting (Abera et al. 2022). The low incidence of chopping and mixing AFs with fresh grasses in Wolaita suggests this practice may be less feasible or less prioritised, possibly due to labour constraints or differences in feed resource availability.

The significant interaction effects between location and season highlight the need to tailor feed management interventions to local conditions. Promoting appropriate feed processing techniques according to seasonal feed availability can optimise utilisation of alternative feed resources and improve smallholder dairy productivity.

### 4.8 Enhancing Smallholder Dairy Productivity and Animal Health Through Alternative Feed Supplementation

The near‐universal recognition of increased milk yield due to supplementation with alternative feeds underscores their vital role in enhancing dairy productivity in smallholder systems (Table 8). This aligns with recent studies emphasising the effectiveness of indigenous and locally available feed resources in improving milk output regardless of season (Bekele et al. 2022; Negash et al. 2023).

Improvements in body condition, milk composition, and milk appearance reported by farmers are indicative of enhanced nutritional status and feed quality provided by AFs. These findings corroborate recent research highlighting that strategic supplementation supports both quantity and quality of milk, thereby improving farmer livelihoods (Dida et al. 2021; Lemma et al. 2023).

The consistent reporting of essential nutrient provision throughout wet and dry seasons suggests that AFs serve as reliable nutrient sources, buffering the impacts of seasonal forage scarcity. This is especially important in mixed crop‐livestock systems vulnerable to climatic variability (Teshome et al. 2021).

The significant difference in perceived improved animal health between Wolaita and Gamo zones may reflect regional differences in management practices, disease prevalence, or the specific types of AFs used. Enhanced health outcomes have been linked to the bioactive compounds and secondary metabolites present in some indigenous plants used as supplements (Abera et al. 2022; Negash et al. 2023). Further research could explore the pharmacological benefits of these species in greater depth.

Overall, the findings highlight the multifaceted benefits of AFs in sustaining and improving milking cow productivity, body condition, and health, emphasising their integral role in smallholder dairy production in Gamo and Wolaita zones of southern Ethiopia.

## 5 Conclusion

The findings reveal a socio‐economically homogeneous population facing common challenges, though occupational differences between regions necessitate tailored interventions, agricultural support in Gamo and livelihood diversification in Wolaita, Ethiopia. A critical need exists to strengthen feed supply chains and enhance on‐farm production through expanded extension services: community feed banks, cooperative development, and decentralised feed hubs. While local knowledge of alternative feeds offers valuable insights, further biochemical analysis and feeding trials are required to validate the efficacy of lesser‐known species. Sustainable livestock production can be improved by integrating indigenous and improved forage varieties alongside feed conservation techniques such as silage and haymaking. Seasonal feed management strategies must prioritise multipurpose species to ensure year‐round availability. Location‐specific extension programs should align feeding practices with local resources while promoting feed processing methods to optimise productivity. Ultimately, adopting alternative supplemental feeds can enhance milk yields, animal health, and livelihood resilience, highlighting the importance of targeted awareness campaigns to facilitate broader uptake. These evidence‐based recommendations provide a pathway for addressing feed scarcity and advancing sustainable dairy production in smallholder systems. By implementing these recommendations, smallholder dairy systems can achieve greater resilience and productivity while reducing reliance on costly, externally sourced feeds. This approach supports sustainable livestock production and improves farmers' livelihoods.

### 5.1 Recommendations

In light of the findings, several recommendations are proposed to enhance the use and sustainability of indigenous alternative supplemental feeds:

1. **Promotion of local feed resources**: Encourage the systematic use of locally available fodder and herb species, particularly in regions with high feed costs and shortages, to reduce dependency on external inputs while maintaining nutritional adequacy.
2. **Multiplication and distribution of planting materials**: Establish seed banks and community‐based nurseries to facilitate the propagation and wider distribution of high‐potential fodder and herbaceous species, ensuring farmers have reliable access to quality planting materials.
3. **Knowledge transfer and extension services**: Strengthen agricultural extension services to disseminate best practices in feed utilisation, conservation techniques, and nutritional management, emphasising the benefits and practical integration of indigenous feeds into livestock diets.
4. **Policy and institutional support**: Policymakers should recognise the value of indigenous feed resources and allocate resources for their conservation and sustainable use, while developing institutional frameworks to support long‐term feed security initiatives.

### 5.2 Future Studies

#### 5.2.1 Agronomic Research and Evaluation

Further research is needed to evaluate the agronomic performance, productivity, and adaptability of promising indigenous species across different agro‐ecological zones to identify the most suitable and high‐yielding varieties for targeted promotion.

## Author Contributions

Asrat Ayza conceptualised the idea of the study, curated the data, performed formal analysis and investigation, designed methodology, administered the project, and wrote the original draft. Yisehak Kechero curated the data, performed formal analysis and investigation, designed methodology, administered the project, and performed data analysis. Ajebu Nurfeta performed a review, performed formal analysis, compiled the report, and edited the manuscript. Dereje Andualem performed a review, performed formal analysis, compiled the report, and edited the manuscript.

## Funding

The study was funded by Arba Minch University with the grant allowance letter referenced as DPC/453/16.

## Ethics Statement

The authors confirm that the journal ethical rules, as outlined on the author guidelines page were followed. However, the study did not use animals for direct experimentation but rather field observation, field measurement, and questionnaire survey. Data collection was carried out following a verbal agreement with the agricultural and local administration offices and dairy farmers.

## Conflicts of Interest

The authors declare no conflicts of interest.

## Consent to Participate

Everyone in the study agreed to take part after getting all the info. Before we gathered data, we told each farmer in their own language about what the study was about – like, why we were doing it, how we'd do it, and that they didn't have to participate if they didn't want to. The study looked at how they use different feeds throughout the year. As per Arba Minch University/Wolaita Sodo University's rules, if someone couldn't read well, we read the info to them and got their thumbprint or said‐yes in voice in front of someone else. Also, we promised they could quit whenever they wanted without getting in trouble, and that their info would be kept secret.

## Data Availability

The data that support the findings of this study are available from the corresponding author upon reasonable request.
